# Supplementary material for: Machine Learning Models Integrating Dietary Indicators Improve the Prediction of Progression from Prediabetes to Type 2 Diabetes Mellitus
Source: Nutrients. 2025 Mar 8;17(6):947. doi: 10.3390/nu17060947 (PMC11945017; doi:10.3390/nu17060947)

Supplementary Table S1. Overview of missingness in 32 baseline variables for prediabetic patients (n=2215).

Variables	N_missing	Complete_rate
Age	0	1.000
Gender	0	1.000
Education levels	0	1.000
Marriage	0	1.000
Income levels	0	1.000
Smoking status	0	1.000
Drinking status	0	1.000
Physical activity	0	1.000
PSQI	543	0.755
HTN	2	0.999
Family history of type 2 diabetes mellitus	0	1.000
Cancer	4	0.998
Kidney failure	3	0.999
FPG	0	1.000
TC	1	1.000
TG	18	0.992
HDL-C	2	0.999
LDL-C	0	1.000
SBP	2	0.999
DBP	2	0.999
Heart rate	2	0.999
BMI	8	0.996
WC	2	0.999
Refined grains	1	1.000
Red meat	0	1.000
White meat	0	1.000
Eggs	1	1.000
Fruits	1	1.000
Vegetables	0	1.000
Beans	0	1.000
Nuts	0	1.000
Whole grains	1	1.000

Supplementary Table S2. Comparison before and after missing value imputation.

Variables	Before	After	*P* value
Refined grains [g/day, *M* (P_{25}, P_{75})]	400(300,500)	400(300,500)	0.989
Eggs [g/day, *M* (P_{25}, P_{75})]	62.50(17.86,62.50)	62.50(17.86,62.50)	0.995
Fruits [g/day, *M* (P_{25}, P_{75})]	85.71(28.57,200.00)	85.71(28.57,200.00)	0.997
Whole grains [g/day, *M* (P_{25}, P_{75})]	50.00(14.29,100.00)	50.00(14.29,100.00)	0.998
PSQI [*M* (P_{25}, P_{75})]	3(2,5)	3(2,5)	0.810
HTN, n (%)			1.000
No	1160(52.42%)	1162(52.46%)	
Yes	1053(47.58%)	1053(47.54%)	
Cancer, n (%)			
No	2188(98.96%)	2192(98.96%)	1.000
Yes	23(1.04%)	23(1.04%)	
Kidney failure, n (%)			
No	2210(99.91%)	2213(99.91%)	1.000
Yes	2(0.09%)	2(0.09%)	
TC [mmol/L, *M* (P_{25}, P_{75})]	5.00(4.40,5.73)	5.00(4.40,5.73)	0.996
TG [mmol/L, *M* (P_{25}, P_{75})]	1.53(1.10,2.31)	1.53(1.11,2.32)	0.816
HDL-C [mmol/L, *M* (P_{25}, P_{75})]	1.24(1.06,1.46)	1.24(1.06,1.46)	0.989
SBP [mmHg, *M* (P_{25}, P_{75})]	132(119,146)	132(119,146)	0.990
DBP [mmHg, *M* (P_{25}, P_{75})]	81(74,90)	81(74,90)	0.967
Heart rate [time/minutes, *M* (P_{25}, P_{75})]	77(69,85)	77(69,85)	0.989
BMI [kg/m^2, *M* (P_{25}, P_{75})]	25.91(23.73,28.25)	25.91(23.73,28.24)	1.000
WC [cm, *M* (P_{25}, P_{75})]	88.00(81.50,94.90)	88.10(81.50,94.95)	0.968

Supplementary Table S3. Univariate logistic regression analysis results based on traditional indicators.

Variables	OR (95%CI)	Z value	*P* value
Age	1.014 (1.003, 1.026)	2.546	**0.011**
Gender1	0.879 (0.699, 1.102)	-1.113	0.266
Education levels1	0.821 (0.657, 1.025)	-1.739	0.082
Marriage1	1.076 (0.744, 1.524)	0.402	0.687
Income levels2	0.890 (0.685, 1.154)	-0.878	0.380
Income levels3	0.722 (0.545, 0.953)	-2.285	**0.022**
Smoking status2	1.185 (0.826, 1.670)	0.947	0.344
Smoking status3	0.817 (0.596, 1.103)	-1.289	0.197
Drinking status2	1.128 (0.656, 1.848)	0.459	0.646
Drinking status3	0.920 (0.690, 1.214)	-0.577	0.564
Physical activity2	0.834 (0.433, 1.668)	-0.531	0.596
Physical activity3	0.943 (0.548, 1.731)	-0.201	0.841
PSQI	0.981 (0.939, 1.023)	-0.878	0.380
HTN1	0.990 (0.792, 1.236)	-0.091	0.928
Family history of type 2 diabetes mellitus1	1.417 (0.870, 2.225)	1.461	0.144
Cancer1	1.372 (0.451, 3.464)	0.622	0.534
Kidney failure1	4.933 (0.195, 124.931)	1.128	0.259
FPG	4.727 (3.075, 7.312)	7.030	**<0.001**
TC	0.787 (0.704, 0.877)	-4.276	**<0.001**
TG	1.108 (1.028, 1.191)	2.731	**0.006**
HDL-C	0.457 (0.316, 0.654)	-4.222	**<0.001**
LDL-C	0.883 (0.777, 1.001)	-1.931	0.054
SBP	1.000 (0.994, 1.006)	0.067	0.947
DBP	0.999 (0.990, 1.009)	-0.183	0.855
Heart rate	0.993 (0.984, 1.003)	-1.347	0.178
BMI	1.037 (1.005, 1.069)	2.293	**0.022**
WC	1.012 (1.001, 1.023)	2.087	**0.037**

Supplementary Table S4. Coefficients for the predictive variables after LASSO regression.

Variables	Coefficients
Age	0.016
Income levels	-0.138
FPG	1.439
TC	-0.269
TG	0.106
HDL-C	-0.319
BMI	0.030

Supplementary Table S5. Variance inflation factor (VIF) of variables

Variables	VIF
Age	1.1157
Income levels	1.0694
FPG	1.0128
TC	1.2375
TG	1.5195
HDL-C	1.5436
BMI	1.1328

Supplementary Table S6. Comparison of model prediction performance based on traditional indicators

Model	AUC (95%CI)	ACC (95%CI)	SPEC (95%CI)	REC (95%CI)	PREC (95%CI)	F1 (95%CI)
LR	0.723 (0.699,0.748)	0.670 (0.646,0.692)	0.666 (0.628,0.700)	0.674 (0.640,0.706)	0.674 (0.641,0.709)	0.674 (0.647,0.701)
SVM	0.787 (0.763,0.810)	0.719 (0.696,0.741)	0.683 (0.649,0.715)	0.754 (0.721,0.784)	0.709 (0.675,0.740)	0.731 (0.706,0.756)
RF	0.853 (0.834,0.872)	**0.779 (0.757,0.798)**	0.724 (0.692,0.756)	**0.834 (0.806,0.860)**	0.755 (0.725,0.783)	**0.793 (0.772,0.811)**
XGBoost	**0.854 (0.835,0.872)**	0.776 (0.754,0.796)	**0.772 (0.742,0.803)**	0.780 (0.752,0.810)	**0.778 (0.749,0.806)**	0.779 (0.753,0.801)

LR, logistic regression; SVM, support vector machine; RF, random forest; XGBoost, extreme gradient boosting; AUC, area under the curve; ACC, accuracy; SPEC, specificity; REC, recall; PREC, precision; F1, F1 score.

Supplementary Table S7. Dietary indicators of prediabetic (n=2215) patients based on their glycemic status at follow-up

Variables	Regressed to NGT (n=1361)	Remained as prediabetes (n=480)	Progressed to T2DM (n=374)	*P* values
Refined grains [g/day, *M* (P_{25}, P_{75})]	400(300,500)	400(300,500)	400(300,500)	**<0.001**
Red meat [g/day, *M* (P_{25}, P_{75})]	14.29(6.67,35.71)	14.29(6.67,37.50)	15.83(6.67,35.71)	0.815
White meat [g/day, *M* (P_{25}, P_{75})]	5.00(0.68,14.29)	5.48(1.58,14.29)	6.67(0.82,16.67)	0.185
Eggs [g/day, *M* (P_{25}, P_{75})]	62.50(18.75,62.50)	62.50(17.86,62.50)	62.50(17.86,62.50)	**0.012**
Fruits [g/day, *M* (P_{25}, P_{75})]	85.71(28.57,200)	100(28.57,200)	100(28.57,200)	0.774
Vegetables [g/day, *M* (P_{25}, P_{75})]	250(150,400)	250(200,450)	250(200,400)	**0.019**
Beans [g/day, *M* (P_{25}, P_{75})]	16.67(6.67,42.86)	16.67(4.11,35.71)	16.67(4.33,42.86)	0.266
Nuts [g/day, *M* (P_{25}, P_{75})]	7.14(0.00,21.43)	6.85(0.00,21.43)	6.67(0.07,16.67)	0.851
Whole grains [g/day, *M* (P_{25}, P_{75})]	50(16.67,100)	50(13.33,100)	33.33(8.33,83.33)	**<0.001**

NGT, normal glucose tolerance; T2DM, type 2 diabetes mellitus.

Supplementary Table S8. Comparison of model prediction performance evaluation metrics based on traditional indicators and dietary indicators

Model	AUC (95%CI)	ACC (95%CI)	SPEC (95%CI)	REC (95%CI)	PREC (95%CI)	F1 (95%CI)
LR	0.732 (0.706,0.758)	0.671 (0.646,0.693)	0.665 (0.629,0.696)	0.677 (0.641,0.709)	0.674 (0.640,0.706)	0.675 (0.648,0.703)
SVM	0.901 (0.885,0.917)	0.828 (0.808,0.847)	0.816 (0.788,0.844)	**0.839 (0.813,0.864)**	0.823 (0.797,0.850)	0.831 (0.810,0.852)
RF	0.920 (0.906,0.933)	0.845 (0.826,0.864)	0.874 (0.848,0.898)	0.817 (0.789,0.844)	0.869 (0.845,0.893)	0.842 (0.821,0.864)
XGBoost	**0.929 (0.916,0.942)**	**0.868 (0.849,0.886)**	**0.900 (0.879,0.920)**	0.836 (0.807,0.863)	**0.895 (0.873,0.916)**	**0.865 (0.845,0.884)**

LR, logistic regression; SVM, support vector machine; RF, random forest; XGBoost, extreme gradient boosting; AUC, area under the curve; ACC, accuracy; SPEC, specificity; REC, recall; PREC, precision; F1, F1 score.

Supplementary Table S9. Delong test for changes in model AUC before and after adding dietary indicators

Model	Before	After	Z values	*P* values
LR	0.723	0.732	-1.290	0.197
SVM	0.787	0.901	-12.553	**<0.001**
RF	0.853	0.920	-9.207	**<0.001**
XGBoost	0.854	0.929	-8.904	**<0.001**

Supplementary Table S10. Comparison of model prediction performance stratified by gender (group validation)

Group	Model	AUC	ACC	SPEC	REC	PRE	F1
Male	LR	0.710<br>(0.660,0.761)	0.653<br>(0.608,0.6971)	0.663<br>(0.612,0.716)	0.632<br>(0.555,0.708)	0.490<br>(0.420,0.555)	0.552<br>(0.483,0.618)
	SVM	**0.867**<br>**(0.830,0.903)**	0.811<br>(0.775,0.844)	0.828<br>(0.785,0.872)	**0.776**<br>**(0.712,0.840)**	0.698<br>(0.627,0.767)	0.735<br>(0.675,0.789)
	RF	0.866<br>(0.826,0.901)	0.813<br>(0.780,0.849)	0.886<br>(0.847,0.921)	0.671<br>(0.603,0.753)	0.750<br>(0.680,0.822)	0.708<br>(0.649,0.767)
	XGBoost	0.859<br>(0.817,0.900)	**0.833**<br>**(0.797,0.866)**	**0.902**<br>**(0.867,0.933)**	0.697<br>(0.618,0.770)	**0.785**<br>**(0.714,0.854)**	**0.739**<br>**(0.680,0.794)**
Female	LR	0.739<br>(0.704,0.768)	0.679<br>(0.649,0.706)	0.666<br>(0.623,0.705)	0.688<br>(0.652,0.725)	0.739<br>(0.702,0.775)	0.713<br>(0.683,0.741)
	SVM	0.908<br>(0.888,0.927)	0.835<br>(0.813,0.857)	0.807<br>(0.770,0.844)	0.855<br>(0.827,0.881)	0.859<br>(0.832,0.888)	0.857<br>(0.836,0.879)
	RF	0.933<br>(0.917,0.946)	0.859<br>(0.837,0.881)	0.865<br>(0.833,0.895)	0.855<br>(0.827,0.882)	0.897<br>(0.872,0.922)	0.876<br>(0.856,0.895)
	XGBoost	**0.947**<br>**(0.934,0.959)**	**0.883**<br>**(0.862,0.902)**	**0.898**<br>**(0.868,0.925)**	**0.872**<br>**(0.843,0.898)**	**0.922**<br>**(0.897,0.944)**	**0.896**<br>**(0.877,0.912)**

Supplementary Table S11. Comparison of model prediction performance stratified by region (spatial validation)

Region	Model	AUC	ACC	SPEC	REC	PRE	F1
East	LR	0.754 (0.705,0.804)	0.694 (0.646,0.736)	0.586 (0.509,0.660)	0.759 (0.703,0.807)	0.754 (0.703,0.807)	0.756 (0.716,0.795)
	SVM	0.912 (0.880,0.942)	0.854 (0.819,0.887)	0.784 (0.721,0.853)	**0.896 (0.858,0.931)**	0.874 (0.835,0.911)	0.885 (0.853,0.912)
	RF	**0.933 (0.909,0.954)**	0.859 (0.829,0.891)	0.864 (0.812,0.916)	0.856 (0.813,0.896)	0.913 (0.879,0.945)	0.883 (0.853,0.909)
	XGBoost	0.932 (0.908,0.953)	**0.873 (0.840,0.903)**	**0.877 (0.827,0.923)**	0.870 (0.828,0.907)	**0.922 (0.884,0.955)**	**0.895 (0.868,0.921)**
South	LR	0.739 (0.692,0.787)	0.659 (0.612,0.701)	0.673 (0.603,0.735)	0.646 (0.582,0.705)	0.692 (0.623,0.754)	0.668 (0.614,0.719)
	SVM	0.920 (0.891,0.945)	0.831 (0.793,0.864)	0.834 (0.779,0.888)	0.827 (0.780,0.878)	0.850 (0.803,0.896)	0.839 (0.801,0.873)
	RF	0.930 (0.904,0.951)	0.847 (0.812,0.880)	0.869 (0.822,0.913)	0.827 (0.776,0.874)	0.878 (0.833,0.921)	0.852 (0.817,0.884)
	XGBoost	**0.940 (0.915,0.960)**	**0.875 (0.842,0.906)**	**0.915 (0.874,0.952)**	**0.841 (0.790,0.889)**	**0.918 (0.880,0.952)**	**0.878 (0.843,0.909)**
West	LR	0.698 (0.598,0.780)	0.677 (0.598,0.744)	0.667 (0.529,0.795)	0.681 (0.598,0.767)	0.819 (0.733,0.895)	0.744 (0.674,0.809)
	SVM	0.890 (0.830,0.939)	0.829 (0.768,0.884)	0.824 (0.706,0.922)	0.832 (0.757,0.896)	0.913 (0.856,0.962)	0.870 (0.819,0.914)
	RF	0.910 (0.859,0.951)	0.866 (0.811,0.915)	0.804 (0.692,0.913)	0.894 (0.837,0.946)	0.910 (0.855,0.958)	0.902 (0.858,0.938)
	XGBoost	**0.965 (0.937,0.986)**	**0.909 (0.866,0.951)**	**0.882 (0.796,0.962)**	**0.920 (0.867,0.967)**	**0.945 (0.899,0.983)**	**0.933 (0.896,0.966)**
North	LR	0.667 (0.618,0.718)	0.654 (0.605,0.695)	0.691 (0.639,0.741)	0.568 (0.482,0.648)	0.446 (0.374,0.522)	0.500 (0.429,0.561)
	SVM	0.861 (0.825,0.898)	0.801 (0.764,0.836)	0.817 (0.772,0.859)	**0.765 (0.691,0.836)**	0.647 (0.570,0.721)	0.701 (0.641,0.760)
	RF	0.867 (0.828,0.905)	0.824 (0.790,0.857)	0.890 (0.855,0.926)	0.674 (0.590,0.754)	0.730 (0.650,0.802)	0.701 (0.636,0.765)
	XGBoost	**0.876 (0.835,0.913)**	**0.848 (0.813,0.880)**	**0.910 (0.876,0.942)**	0.705 (0.623,0.782)	**0.775 (0.697,0.844)**	**0.738 (0.677,0.795)**

Supplementary Table S12. Model parameter settings based on traditional indicators and dietary indicators

Model	AUC
LR	penalty='l2'<br>C=0.013
SVM	gamma=0.1<br>C=0.9<br>kernel='rbf'
RF	max_depth=14<br>max_features=1.0<br>n_estimators=500
XGBoost	learning_rate=0.09<br>max_depth=3<br>n_estimators=500

Supplementary Figure S1. The calibration curve of T2DM prediction models based on traditional indicators

A

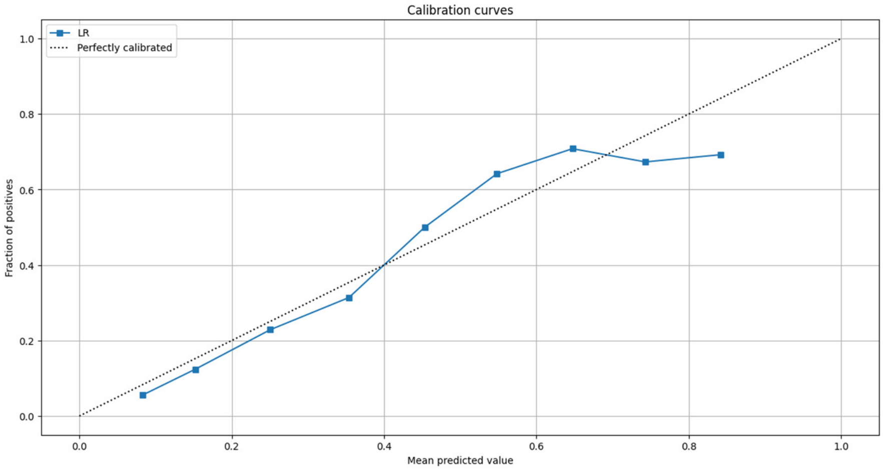

B

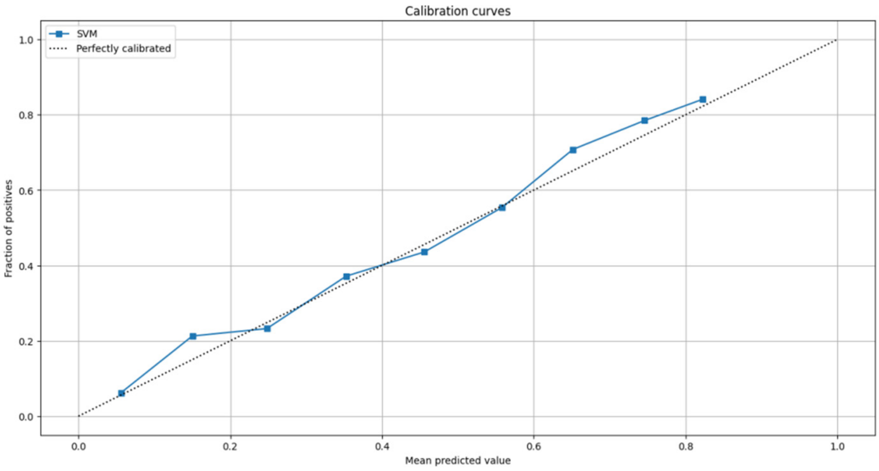

C

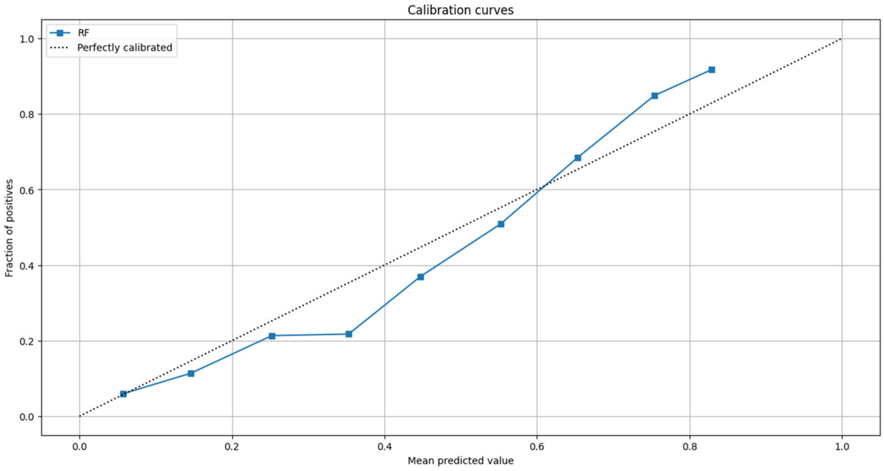

D

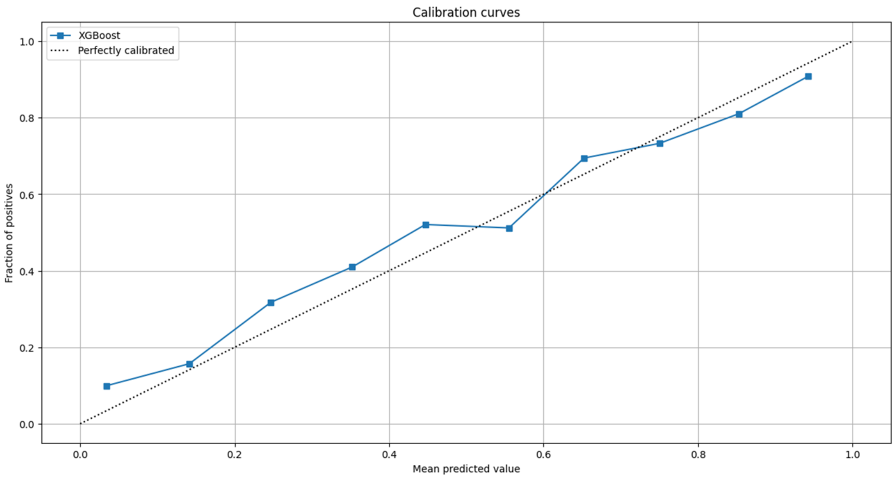
Calibration curves
XGBoost
Perfectly calibrated
Fraction of positives
Mean predicted value
0.0
0.2
0.4
0.6
0.8
1.0

Supplementary Figure S2. The calibration curve of T2DM prediction models based on traditional and dietary indicators

A

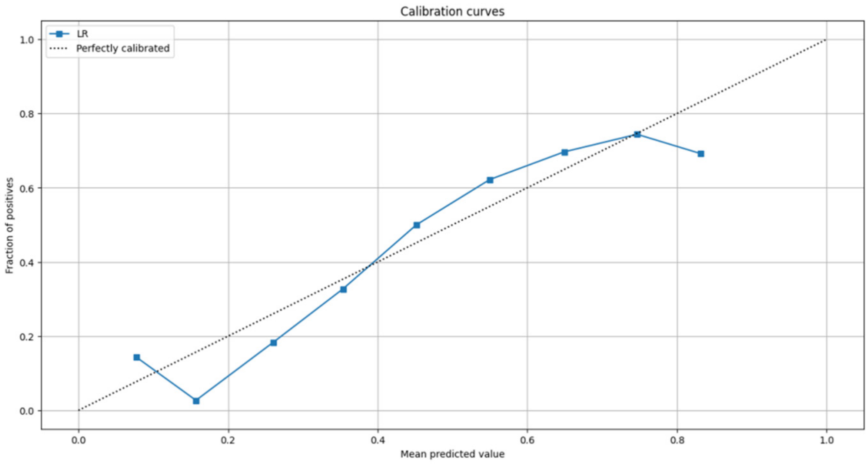

B

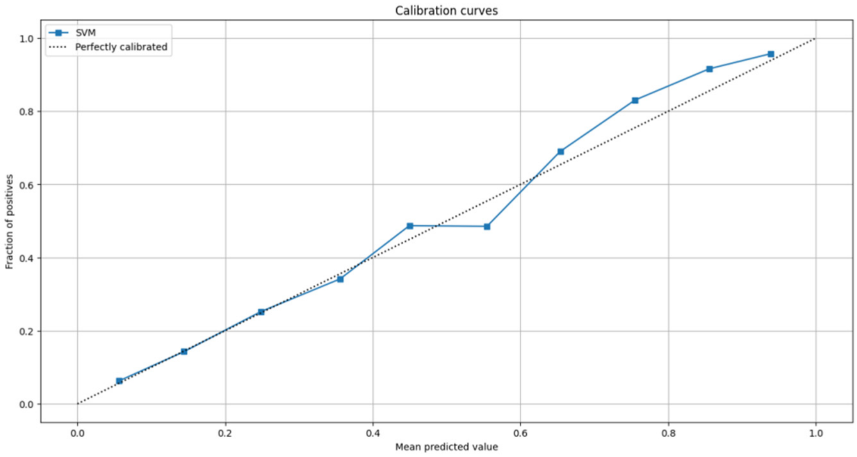

C

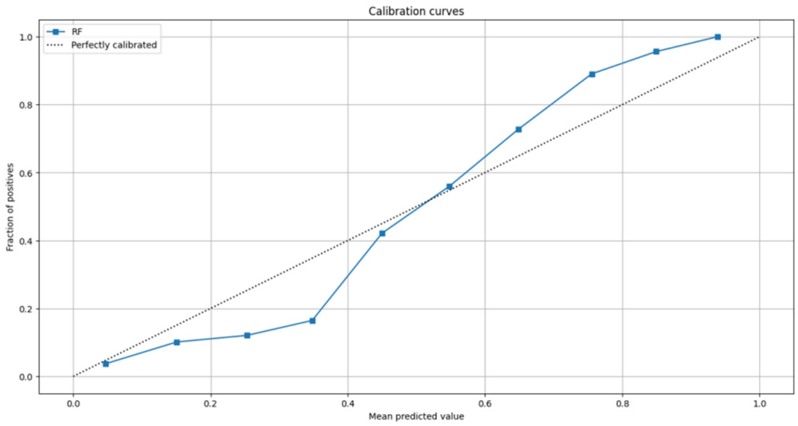

D

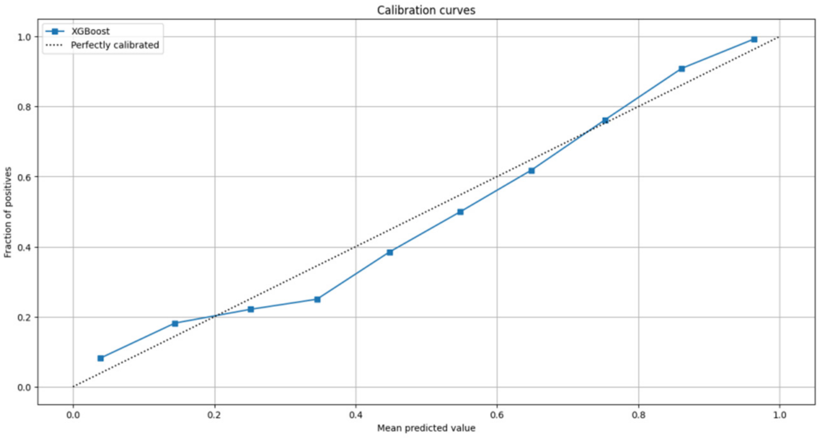
Calibration curves
XGBoost
Perfectly calibrated
Fraction of positives
Mean predicted value
0.0
0.2
0.4
0.6
0.8
1.0

Supplementary Figure S3. The decision curve analysis (DCA) of the T2DM prediction model based on traditional and dietary indicators.

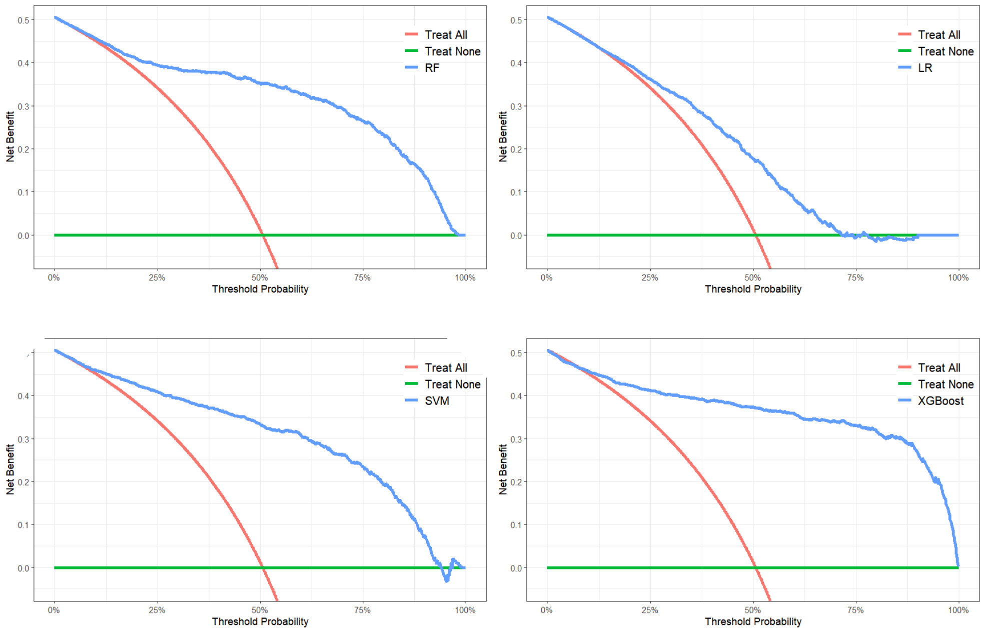

Supplement: Supplementary file 1 [file nutrients-17-00947-s001.zip › nutrients-3497475-supplementary.pdf]
